# Supplementary material for: Verticillium dahliae Inoculation and in vitro Propagation Modify the Xylem Microbiome and Disease Reaction to Verticillium Wilt in a Wild Olive Genotype
Source: Front Plant Sci. 2021 Mar 3;12:632689. doi: 10.3389/fpls.2021.632689 (PMC7966730; doi:10.3389/fpls.2021.632689)
Supplement: Supplementary Figure 1 — Effect of inoculation with the defoliating pathotype of Verticillium dahliae in “Picual,” “Ac-15,” and “Ac-18,” plants obtained using in vitro-standard, in vitro-adapted, and nursery propagation methods. “Picual” and “Ac-15” were used as positive control to determine the inoculation success and the development of the disease. Note the defoliation of green leaves observed for in vitro-standard propagated “Ac-18” plants. Picture was taken 44 days after inoculation with the pathogen. [file Data_Sheet_1.pdf]

Supplementary Material

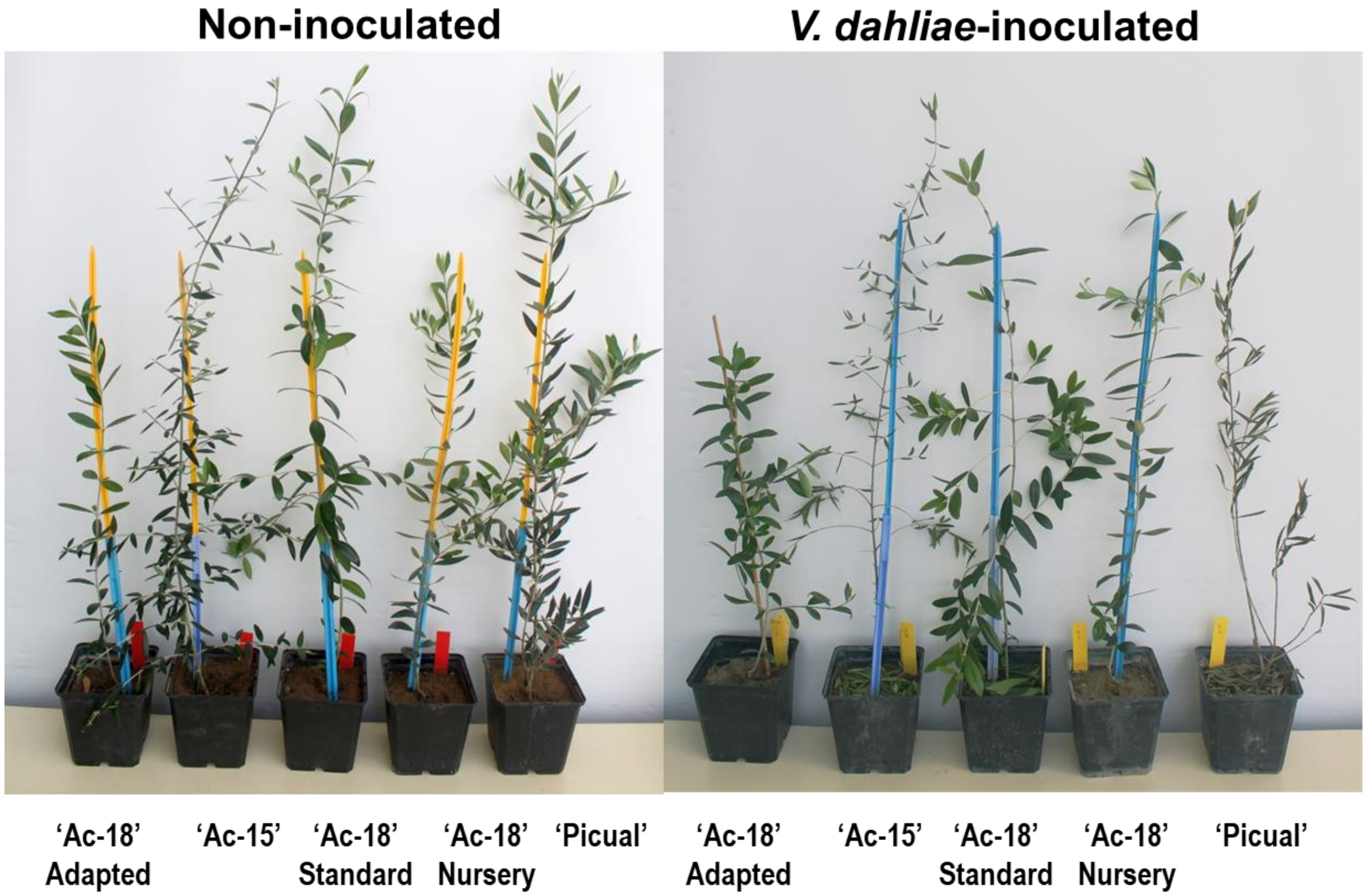

Figure S1. Effect of inoculation with the defoliating pathotype of *Verticillium dahliae* in 'Picual', 'Ac-15' and 'Ac-18', plants obtained using *in vitro*-standard, *in vitro*-adapted and nursery propagation methods. 'Picual' and 'Ac-15' were used as positive control to determine the inoculation success and the development of the disease. Note the defoliation of green leaves observed for *in vitro*-standard propagated 'Ac-18' plants. Picture was taken 44 days after inoculation with the pathogen.

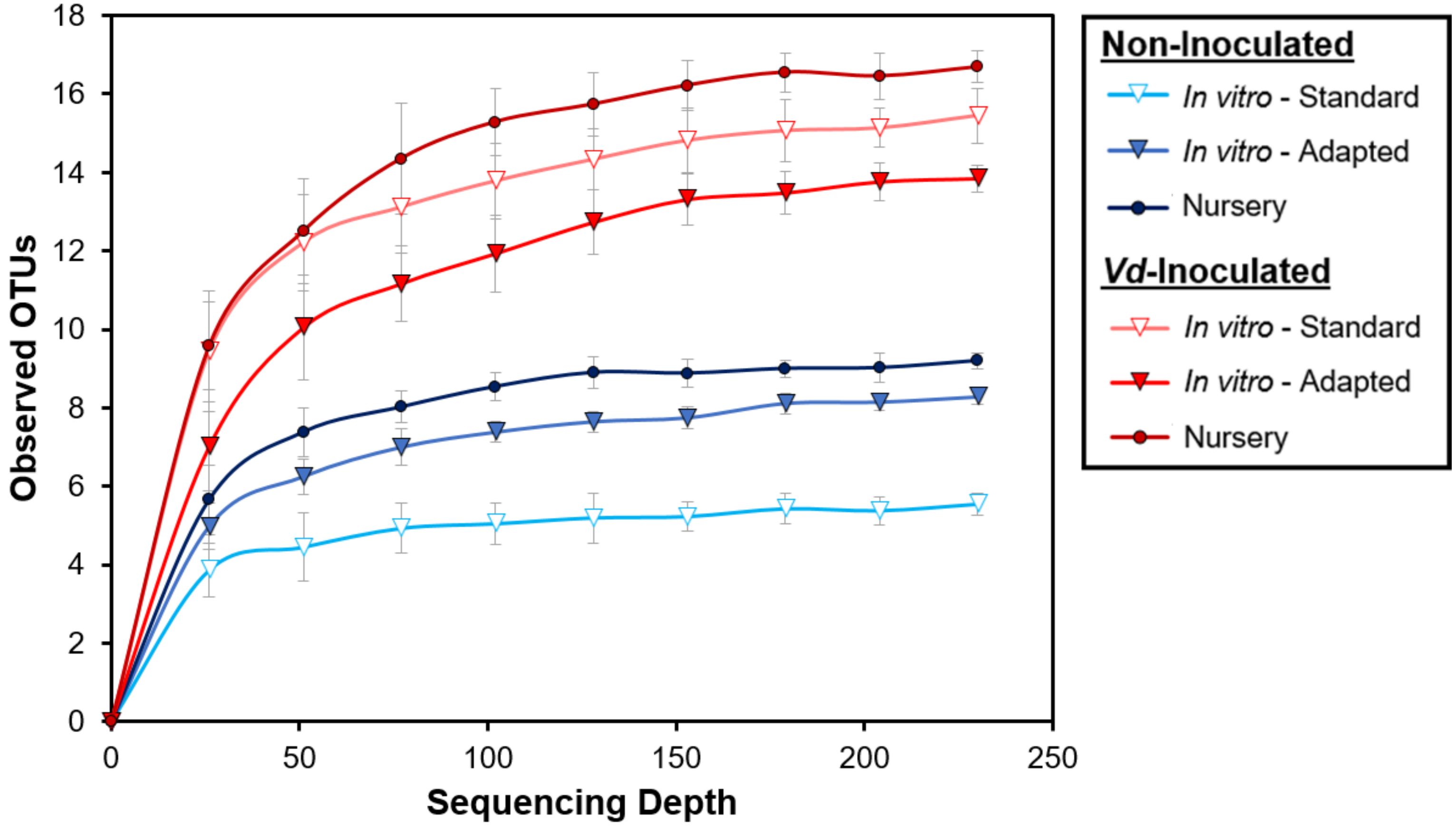

Figure S2. Richness rarefaction curves at OTU taxonomic level in olive xylem from *Verticillium dahliae* (*Vd*)-inoculated and non-inoculated (NI) ‘Ac-18’ plants following *in vitro* (standard and adapted) and nursery propagation methods. Error bars represent standard error of six values.

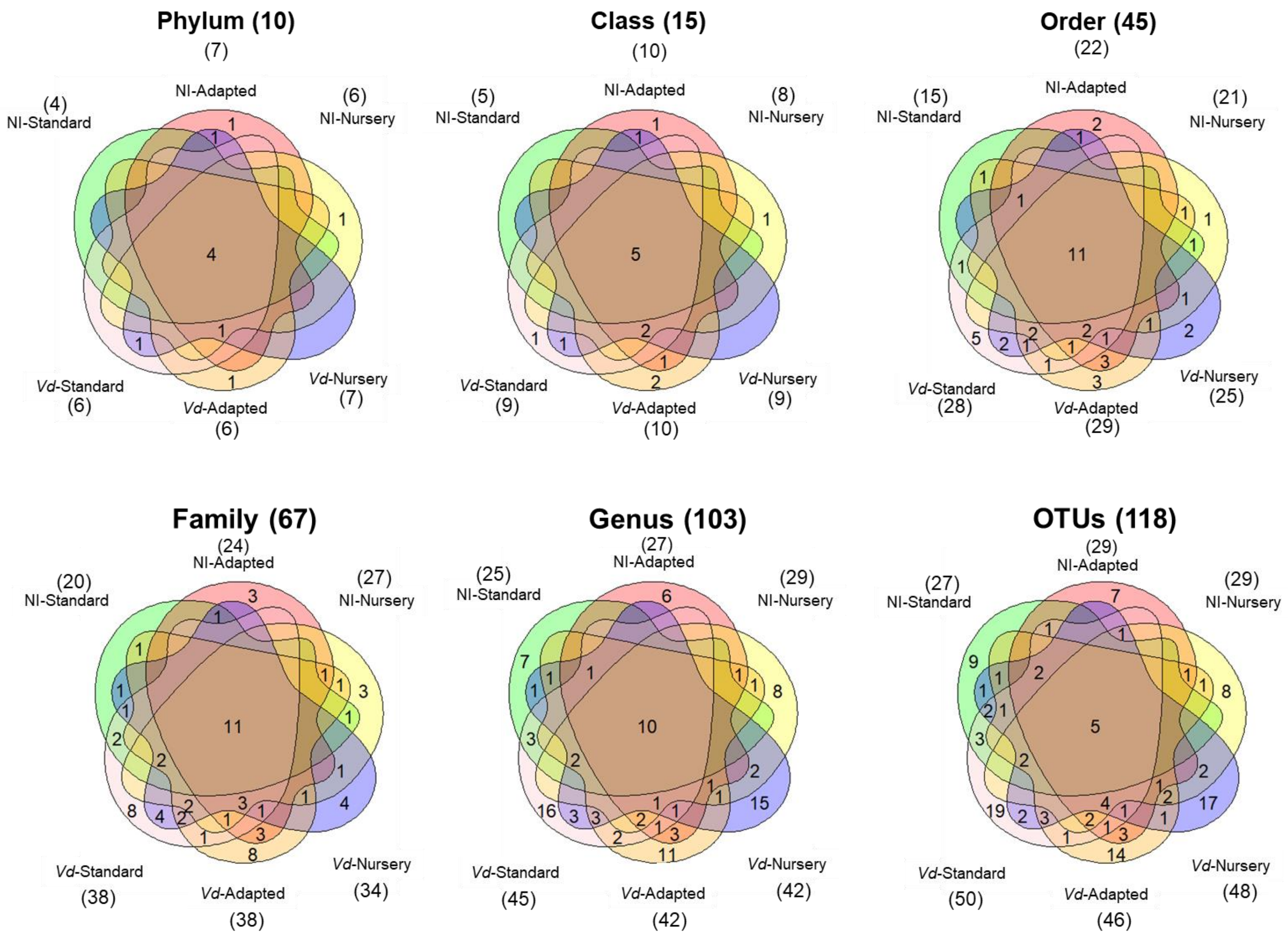

Figure S3. General prevalence Venn diagram showing the unique and shared bacteria at different taxonomy ranks in olive xylem from *Verticillium dahliae* (*Vd*)-inoculated and non-inoculated (NI) 'Ac-18' plants following *in vitro* (standard and adapted) and nursery propagation methods.

Table S1. The Scheirer–Ray–Hare test showing H and *P*-value on Richness, Shannon, and Evenness alpha diversity indexes to assess the effects of the inoculation treatment, the plant propagation method (*in vitro*-standard, *in vitro*-adapted and nursery) and their interaction.

**Diversity index**	**Treatment**	**df**	**Sum of squares**	**H**	***P*-value**
Richness	Inoculation treatment	1	1032.27	9.903	**0.0017**
	Propagation method	2	95.82	0.919	0.6315
	Inoculation x propagation	2	57.54	0.552	0.7588
	Residuals	29	2358.37		
Shannon	Inoculation treatment	1	1674.62	15.949	< **0.0001**
	Propagation method	2	322.04	3.067	0.2158
	Inoculation x propagation	2	90.87	0.865	0.6488
	Residuals	29	1482.47		
Evenness	Inoculation treatment	1	263.53<br>2.5098	2.5098	0.11314
	Propagation method	2	1133.02	10.7907	**0.00454**
	Inoculation x propagation	2	91.08	0.8675	0.64809
	Residuals	29			

Table S2. ADONIS test showing R^2 and *P*-values to assess the significance of the inoculation treatment and the plant propagation method and their interaction

Treatment	df	Sequential Sums of Squares	Mean Squares	F	partial R^2	*P*-value
Inoculation treatment	1	0.0514	0.0514	5.3911	0.1115	**0.004**
Propagation method	2	0.1235	0.0617	6.4780	0.2680	**0.001**
Inoculation x Propagation	2	0.0286	0.0143	1.5008	0.0621	0.175
Residuals	27	0.2573	0.0095		0.5584	
Total	32	0.4610			1	
